# Evaluation of the Relationship Between Facial Hue and Blood Pressure Fluctuations in Hemodialysis Patients

**DOI:** 10.1155/ijbi/8387784

**Published:** 2026-08-31

**Authors:** Kosuke Oiwa, Takehiro Okama, Satoshi Suzuki, Yoshitaka Maeda, Akio Nozawa

**Affiliations:** ^1^ Department of Information and Management Systems Engineering, Nagaoka University of Technology, Nagaoka, Niigata, Japan, nagaokaut.ac.jp; ^2^ Medical Corporation Credo, Sato Clinic, Chiba, Chiba, Japan; ^3^ Department of Clinical Engineering, Kanagawa Institute of Technology, Atsugi, Kanagawa, Japan, kait.jp; ^4^ Medical Simulation Center, Jichi Medical University, Shimotsuke, Tochigi, Japan, jichi.ac.jp; ^5^ College of Science and Engineering, Aoyama Gakuin University, Sagamihara, Kanagawa, Japan, aoyama.ac.jp

**Keywords:** blood pressure estimation, facial hue information, hemodialysis, L^*^a^*^b^*^ color space, noncontact sensing, RGB color space

## Abstract

**Objective:**

In hemodialysis, hypotension may occur due to fluid removal, but traditional intermittent blood pressure measurements risk missing its onset. We are aimed to enabling noninvasive and continuous blood pressure monitoring in patients undergoing hemodialysis based on the spatial features of facial visible images. Facial visible images contain not only skin chromaticity information but also facial expression–related variations. To improve estimation, facial color components are separated from expressions, analyzed for their relationship with blood pressure fluctuations, and used to build an individual estimation model.

**Methods:**

Facial landmark coordinates were extracted, and eight facial regions of interest (ROIs) were defined on facial images of hemodialysis patients. Mean and differential color values between ROIs served as explanatory variables, whereas mean blood pressure was the dependent variable. Stepwise selection identified key variables for blood pressure estimation, followed by regression modeling.

**Results:**

Applying a linear regression model to the mean color values in each ROI resulted in a higher generalization performance across all subjects. The facial hue components of the a ^∗^ and b ^∗^ values were used as explanatory variables contributing to blood pressure estimation in all subjects, but there were individual differences in the location of ROIs selected as explanatory variables.

**Conclusion:**

Applying a linear regression model to the absolute values of facial hue improved generalization performance, and there are individual differences in the relationship between facial hue components and blood pressure fluctuations. This study is significant because these findings could contribute to the realization of remote blood pressure monitoring during hemodialysis.

## 1. Introduction

Currently many patients with end‐stage renal disease undergo hemodialysis, a treatment that artificially purifies blood by removing excess fluids and solutes. It has been reported that approximately 40% of these patients experience hypotension, particularly intradialytic hypotension, which is recognized as a risk factor for cardiovascular complications [1]. The causes of hypotension during hemodialysis include fluid removal, vasodilation caused by dialysate components, and osmotic pressure reduction due to solute removal. During hemodialysis, fluid removal and solute elimination must occur within a limited timeframe, causing rapid changes in circulating blood volume. If fluid transfer from interstitial to vascular compartments cannot keep up, hypotension may occur.

Blood pressure is one of the most critical parameters to monitor in hemodialysis patients. In clinical settings, blood pressure is typically measured noninvasively using a cuff wrapped around the upper arm. However, this intermittent method is usually performed every 30 min–1 h, posing the risk of missing the onset of hypotension. Furthermore, the requirement for direct physical contact can compromise comfort and make the process labor‐intensive for medical staff.

These limitations highlight the need for alternative methods that can provide continuous and noncontact blood pressure monitoring. Such approaches would enable real‐time tracking of hemodynamic status without the need for cuffs or frequent manual intervention, potentially improving patient safety and workflow efficiency.

In recent years, several studies have explored the use of deep learning to predict intradialytic hypotension. Many of these studies have developed models to predict hypotension during dialysis by applying AI algorithms to multiple patient variables, including age, body weight, intermittently measured blood pressure values, and dialysis settings [2–5]. Additionally, some studies have attempted to predict hypotension using only time‐series data of heart rate variability obtained from electrocardiograms [6].

Despite these efforts, continuous and noninvasive blood pressure monitoring remains a critical but unsolved challenge in hemodialysis care. Although conventional sensors often rely on direct physical contact, there is increasing interest in developing novel sensing principles that can infer physiological parameters through remote, contact‐free modalities. In this context, sensors are no longer limited to hardware components alone but encompass the integration of signal acquisition, feature extraction, and estimation algorithms into a cohesive sensing system.

Although most existing studies focus on predicting hypotensive states, no research has been conducted on continuous blood pressure monitoring during dialysis. Furthermore, existing prediction methods require a large number of explanatory variables. An ideal blood pressure monitoring method should be continuous, allowing for the early detection of blood pressure fluctuations and enabling timely adjustments to dialysis conditions, such as fluid removal rates, to prevent hypotension. Moreover, implementing a remote blood pressure monitoring system could eliminate the need for cuffs, providing benefits in terms of infection control. Our research group has investigated methods for estimating blood pressure in healthy individuals based on spatial features of facial images captured in the visible, near‐infrared, and infrared bands [7–10]. Facial images in the visible and near‐infrared bands contain chromatic components of the skin, whereas those in the infrared band contain thermal components of the skin. Both skin chromaticity and skin temperature reflect skin blood flow, making facial images valuable for noncontact hemodynamic monitoring. Recently, our research group has initiated a study aimed at monitoring blood pressure during hemodialysis. In related previous research, spatial features were extracted from visible and infrared facial images, and deep learning was used to estimate the mean blood pressure of three hemodialysis patients [11]. However, the accuracy of blood pressure estimation based on spatial features extracted from facial visible images was low. This was attributed to the inclusion of not only skin chromaticity but also facial expressions in the images. Spatial features associated with facial expressions, such as blinking, which are unrelated to blood pressure fluctuations, were extracted, thereby reducing estimation accuracy.

To realize blood pressure monitoring during hemodialysis, it is essential to clearly distinguish between skin chromaticity components and the effects of facial expressions, and to evaluate their relationships with blood pressure fluctuations. In our previous studies, we analyzed chromatic components in the RGB and L ^∗^a ^∗^b ^∗^ color spaces extracted from 13 regions of interest (ROIs) in facial visible images and quantitatively evaluated their causal relationships with subjective sensations related to health conditions [12]. These studies demonstrated the potential for health condition monitoring based on skin chromaticity components while excluding the effects of facial expressions.

This study is an initial study to evaluate facial color and blood pressure variability in dialysis patients, and facial visible images of hemodialysis patients were analyzed to identify specific regions where the facial hue fluctuates significantly due to blood flow changes. The hue components from these regions were extracted, and an individual model for blood pressure estimation was constructed based on these components. This model can estimate blood pressure from the facial hue components derived from a single facial visible image, allowing for unconscious and prompt blood pressure estimation.

Although a commercial camera was employed in this study, our approach represents a step toward creating a novel noncontact sensor system by redefining how optical data from the skin can be transformed into hemodynamic information. The proposed method, which extracts meaningful physiological signals from facial hue variations, serves as a software‐defined sensor layer that can be integrated with various imaging platforms. Future work will aim to incorporate this sensing principle into practical systems, potentially enabling remote blood pressure monitoring through consumer‐grade devices, and contributing to the field of sensor design in biomedical applications.

## 2. Materials and Method

**Figure 1:**
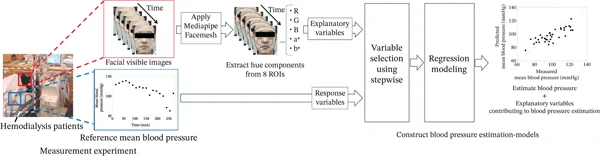
An approach of this study. Regression models for blood pressure estimation were developed using facial hue information of hemodialysis patients, obtained in measurement experiments, as explanatory variables. Reference blood pressure values measured in the same experiments served as the response variable. Key explanatory variables contributing to blood pressure estimation were also identified.

The study approach is shown in Figure 1. In the experiment, facial visible images and reference mean blood pressure values were obtained from patients undergoing hemodialysis. The measured facial visible images were processed using MediaPipe FaceMesh, a machine learning–based library capable of detecting and tracking 468 3D‐facial landmarks in real time, to obtain facial landmark coordinates [13]. Based on this coordinate information, eight ROIs on the face were defined. For each ROI, the average values of red (R), green (G), and blue (B) in the RGB color space, as well as the a ^∗^ and b ^∗^ values representing hue and chroma in the L ^∗^a ^∗^b ^∗^ color space, were calculated as facial color components. In this study, the mean values of R, G, B, a ^∗^, and b ^∗^ for the eight ROIs, as well as the differences in R, G, B, a ^∗^, and b ^∗^ between two ROIs, were used as explanatory variables. The mean blood pressure value measured during the experiment was set as the dependent variable. Stepwise selection was applied to the explanatory and dependent variables to select the explanatory variables contributing to blood pressure estimation. After variable selection, regression modeling was performed based on the selected explanatory variables and the dependent variable, creating an individual model for blood pressure estimation using facial chromatic components. Furthermore, by identifying the explanatory variables contributing to blood pressure estimation, the relationship between facial chromatic components and blood pressure fluctuations was evaluated. The present study was conducted in accordance with the Declaration of Helsinki and was approved by the Ethics Review Committee of Jichi Medical University (Approval No.: 23‐107).

### 2.1. Measurement Experiment

The experimental system consists of a visible camera (C920, Logitech) for facial image capture and a dialysis support system. The camera was positioned 0.5 m from the subject′s face and has a resolution of 1920 × 1080 pixels. A cuff attached to the dialysis support system was placed on the subject′s upper arm to intermittently measure systolic and diastolic blood pressures. The dialysis support systems used varied for each subject. Specifically, DBB‐100NX (Nikkiso) was used for Subjects A–C, and either DCS‐100NX (Nikkiso) or DCS‐200Si (Nikkiso) was used for Subjects D–H. The blood pressure measurement performance of these dialysis support systems is equivalent. The mean blood pressure was calculated by the authors from the measured systolic blood pressure and diastolic blood pressure using the following equation:

(1) Mean blood pressure=diastolic blood pressure+systolic blood pressure−diastolic blood pressure3.

The experiment was conducted in a clinic with a relatively stable room temperature. Image acquisition was performed under daylight‐color fluorescent lighting. The lighting conditions were not intentionally changed during each dialysis session; however, the illuminance and color temperature were not quantitatively measured. The camera was operated using its default settings, and the white balance, exposure, and gain were not manually fixed. No color calibration target was used during image acquisition. During the experiment, the hemodialysis patients were asked to lie on a bed in a supine position. They were allowed to remain in a relaxed state without being forced to maintain a specific posture. Throughout the approximately 4‐h hemodialysis session, visible images including the subject′s face were continuously captured at a frame rate of 30 fps using the visible camera. Systolic and diastolic blood pressures were manually measured approximately every 15 min. A total of eight hemodialysis patients participated in the experiment, comprising five males (Subjects A, B, C, D, and F) and three females (Subjects E, G, and H). Table 1 summarizes the number of experimental sessions and total blood pressure measurements for each subject. Due to differences in body size and weight among the subjects, the duration of each hemodialysis session varied, resulting in differences in the number of blood pressure measurements.

**Table 1:** Number of experimental sessions and total blood pressure measurements for each subject.

Subject	A	B	C	D	E	F	G	H
Experimental sessions	6	10	8	12	13	5	11	10
Blood pressure measurements	126	156	134	200	216	87	200	154

### 2.2. Extraction of Facial Hue Components

In the measurement experiments, visible images including the subject′s face were continuously recorded, whereas blood pressure was measured manually approximately once every 15 min. To construct the blood pressure estimation model using facial hue components described later, it was necessary to use the hue information at the time of blood pressure measurement as data. However, since measuring blood pressure requires a certain amount of time, and there were cases in which the subject′s face was turned sideways during the measurement, preventing the acquisition of facial hue information, the hue information extracted from any visible image measured within 5 min before or after the start of the blood pressure measurement was used as the hue information at the time of measurement. Images were excluded from the analysis when the face was not oriented sufficiently toward the camera and the required ROIs were not fully visible, or when facial landmark detection failed.

**Figure 2:**
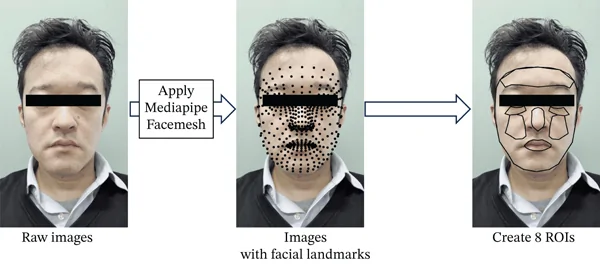
Approach for extracting facial hue components. MediaPipe FaceMesh was applied to visible images to extract landmarks, and eight ROIs were defined based on the landmark coordinates.

The approach for extracting facial hue components is illustrated in Figure 2. MediaPipe FaceMesh, a machine learning–based facial landmark detection library, was applied to the visible images obtained in the measurement experiments, extracting a total of 468 facial landmarks. Based on the coordinates of these landmarks, eight ROIs were defined: the entire face, forehead, right infraorbital, left infraorbital, nose, right cheek, left cheek, and lips. Among these, the right infraorbital, left infraorbital, nose, right cheek, left cheek, and lips are known to exhibit significant hue changes due to variations in skin blood flow. The thin skin of the infraorbital regions makes blood flow changes more visually noticeable. The nose contains numerous capillaries beneath the skin, leading to substantial changes in skin color and temperature due to blood flow fluctuations. The right and left cheeks have relatively high blood flow, resulting in pronounced hue variations. The lips, characterized by thin skin and densely packed capillaries, exhibit particularly large hue changes in response to blood flow fluctuations. On the other hand, the forehead typically exhibits a relatively lower density of superficial capillaries and more uniform vascular distribution compared with other facial regions such as the cheeks or nose. Additionally, the skin on the forehead tends to be thinner and less affected by muscle movements and facial expressions. These anatomical and physiological characteristics result in less hue variation, making the forehead a stable and reliable reference region for calculating relative hue changes. This choice facilitates clearer evaluation of blood flow fluctuations in other facial areas. Table 2 lists the areas of each ROI defined by the facial landmark numbers in MediaPipe FaceMesh. Each ROI corresponds to the area formed by connecting the coordinate numbers shown in Table 2, and the facial landmark numbers within each ROI are listed in a counterclockwise order. For the color information used in this study, both the commonly used RGB color space and the L ^∗^a ^∗^b ^∗^ color space were employed. The RGB color space is a color model intended for display output, and it is characterized by the fact that human perception of color changes differs. On the other hand, the L ^∗^a ^∗^b ^∗^ color space is designed to approximate human vision, allowing for the perception of color differences to be handled equally. In the L ^∗^a ^∗^b ^∗^ color space, the L ^∗^ value represents brightness, whereas the a ^∗^ and b ^∗^ values represent hue and chroma, respectively. In this study, the L ^∗^ component was not included as an explanatory variable because the primary objective was to evaluate blood flow–related changes in facial hue rather than changes in luminance. Since L ^∗^ mainly represents image brightness, it was considered less directly related to physiological changes in skin blood flow than the a ^∗^ and b ^∗^ components. Furthermore, the L ^∗^ component was not used as a quality‐control indicator for assessing lighting conditions or image quality in the present study. Therefore, in this study, the RGB color space values for red (R), green (G), and blue (B), as well as the hue and chroma indicators a ^∗^ and b ^∗^ in the L ^∗^a ^∗^b ^∗^ color space, were used as the hue information. RGB images were converted to the L ^∗^a ^∗^b ^∗^ color space using the color conversion function implemented in OpenCV. No additional color correction, illumination correction, or specific processing to detect or remove specular reflection or halation was applied.

**Table 2:** Areas of each ROI defined by the facial landmark numbers in MediaPipe FaceMesh. Landmark numbers for each ROI are listed in counterclockwise order.

ROI	Facial landmark numbers
Entire face	10, 67, 54, 21, 234, 215, 135, 150, 176, 152, 400, 379, 364, 435, 454, 251, 284, 297
Forehead	63, 105, 66, 107, 9, 336, 296, 334, 293, 298, 284, 332, 297, 338, 10, 109, 67, 103, 54, 68
Right infraorbital	110, 24, 23, 22, 26, 121, 120, 119, 118, 117
Left infraorbital	339, 254, 253, 252, 256, 350, 349, 348, 347, 346
Nose	197, 196, 174, 236, 134, 220, 239, 2, 459, 440, 363, 456, 399, 419
Right cheek	119, 118, 50, 187, 207, 216, 92, 165, 129, 100
Left cheek	329, 358, 391, 322, 436, 427, 411, 280, 347, 348
Lip	62, 91, 181, 84, 17, 314, 405, 321, 375, 409, 269, 267, 0, 37, 39

Furthermore, this study also adopted an evaluation method using relative values of hue information. Facial hue consists of intrinsic skin color and color components related to blood circulation. Skin color associated with blood flow varies with changes in skin blood flow due to blood pressure fluctuations, with greater variation in areas with high vascular density and smaller variation in areas with low vascular density. Based on this characteristic, it was hypothesized that evaluating the difference in hue information between two ROIs would enable the estimation of changes in skin blood flow associated with blood pressure fluctuations.

In fact, our previous study conducted physiological and psychological evaluations using the difference in hue information between two ROIs in facial visible images. Based on these considerations, this study defined the average values of R, G, B, a ^∗^, and b ^∗^ in the eight ROIs (hereinafter referred to as “absolute values”) and the differences in R, G, B, a ^∗^, and b ^∗^ between two ROIs (hereinafter referred to as “relative values”) as evaluation indices for facial hue.

### 2.3. Construction of a Blood Pressure Estimation Model

The construction of the blood pressure estimation model consists of the steps of defining the explanatory and target variables, selecting the explanatory variables, and modeling using a regression model.

The absolute and relative values of facial hue in each ROI were used as explanatory variables, with the mean blood pressure values as the objective variable. The explanatory variables were entered into the regression models using their original numerical scales, without standardization or normalization. It is anticipated that explanatory variables related to facial hue will include not only skin blood flow variations due to blood pressure fluctuations but also hue components resulting from other factors. Stepwise regression based on *p* values was applied to select the explanatory variables that contribute to blood pressure estimation. The method involves starting with an empty model, sequentially adding explanatory variables, calculating their *p* values, and repeatedly removing the least significant variable (the one with the largest *p* value). In this study, the threshold for *p* values in the stepwise method was set at 0.1. This relatively relaxed threshold was chosen to balance sensitivity and specificity in variable selection, particularly due to the relatively small sample size. Using the conventional threshold of 0.05 might have excluded explanatory variables that could still provide valuable information for exploratory modeling in this preliminary study. As described below, four modeling techniques were employed in this study, and the explanatory variables applied to each model were the common ones selected using the aforementioned method.

This study employed linear and nonlinear regression for regression modeling. Linear or nonlinear regression modeling was applied to the selected explanatory variables and the target variable to construct individual models for blood pressure estimation. In this study, the aim was to construct individual models, so the data used for modeling consisted of data from each subject (the data measured in experiments performed a number of times, as indicated in Table 1). The linear regression modeling methods used in this study included multiple regression analysis (hereafter referred to as MRA) and support vector regression with a linear kernel (hereafter referred to as Linear‐SVR). Nonlinear regression modeling methods included support vector regression with an RBF kernel (hereafter referred to as Nonlinear‐SVR) and random forest regression (hereafter referred to as RF). These regression models were implemented in Python using scikit‐learn Version 1.0.2 [14]. MRA was implemented using LinearRegression. Linear‐SVR and Nonlinear‐SVR were implemented using SVR with kernel =  ^“^linear^”^ and kernel =  ^“^rbf,  ^”^ respectively. RF was implemented using RandomForestRegressor with 100 trees and random_state = 42. The remaining parameters were set to their default values.

To evaluate the generalization performance of the models, a threefold cross‐validation was performed. One‐third of the dataset, consisting of the explanatory and target variables for each subject, was used as the test data, whereas the remaining data were used for training. The combinations of test and training data were altered to assess the model′s generalization performance. The evaluation metrics for generalization performance included the correlation coefficient between the measured and estimated mean blood pressure, as well as the root mean squared error (RMSE).

Additionally, evaluation of explanatory variables contributing to blood pressure estimation was also performed. By determining the importance of explanatory variables, the contribution of each variable to blood pressure estimation was assessed. However, the method for determining the importance of explanatory variables differed across regression models. For MRA, the absolute values of the regression coefficients were used, with larger absolute values indicating stronger influence on the dependent variable. For Linear‐SVR, Nonlinear‐SVR, and RF, permutation importance was used to assess the importance of explanatory variables. Permutation importance can capture the nonlinear effects of features, making it applicable to nonlinear regression models [15]. The calculation of permutation importance is as follows. First, the trained model is applied to the test data to calculate the coefficient of determination (${{\text{R}}^{2}}_{\text{original}}$), which is used as the baseline value. Then, one explanatory variable from the test data is randomly shuffled, and the trained model is applied again to calculate the new coefficient of determination (${{\text{R}}^{2}}_{\text{shuffled}}$). If ${{\text{R}}^{2}}_{\text{shuffled}}$ significantly decreases compared with ${{\text{R}}^{2}}_{\text{original}}$, it indicates that the shuffled explanatory variable has high importance. This process is repeated multiple times to determine the importance of each explanatory variable. In the present study, permutation importance was obtained by calculating the difference between ${{\text{R}}^{2}}_{\text{original}}$ and ${{\text{R}}^{2}}_{\text{shuffled}}$, and this procedure was repeated multiple times to determine the importance of each explanatory variable.

### 2.4. Use of Artificial Intelligence

ChatGPT (OpenAI) was used only to improve the English language and readability of the manuscript. The authors reviewed and edited all AI‐assisted text and took full responsibility for the final content. ChatGPT was not used for the study design, data analysis, interpretation of the results, or generation of the scientific conclusions.

## 3. Results

### 3.1. Generalization Performance Evaluation of a Blood Pressure Estimation Model

Table 3 summarizes the differences between the maximum and minimum values of the mean blood pressure obtained across all experiments for each subject. These values correspond to the range of the target variable when constructing individual models for blood pressure estimation. As a result, differences in the mean blood pressure variation were observed among subjects.

**Table 3:** Differences between maximum and minimum mean blood pressure obtained across all experiments for each subject (unit: [mmHg]).

Subject	A	B	C	D	E	F	G	H
Differential value	85.7	57.0	55.7	76.7	94.0	101.7	95.0	48.7

Table 4 presents the coefficient of determination for each subject′s model, as well as the RMSE between the measured and estimated values of mean blood pressure, using absolute values and relative values of facial hue in each modeling method. The results show the mean and standard deviation of the values obtained through threefold cross‐validation.

**Table 4:** Coefficient of determination and RMSE between the measured and estimated values of mean blood pressure for each subject using the absolute and relative values of facial hue. Results show the mean and standard deviation of the values obtained through threefold cross‐validation.

Subject	A	B	C	D	E	F	G	H
Absolute values								
Coefficient of determination								
MRA	0.534 ± 0.203	0.405 ± 0.035	0.282 ± 0.130	0.452 ± 0.016	0.451 ± 0.082	0.583 ± 0.058	0.523 ± 0.056	0.246 ± 0.091
Linear‐SVR	0.558 ± 0.038	0.260 ± 0.139	0.290 ± 0.061	0.263 ± 0.120	0.434 ± 0.059	0.495 ± 0.131	0.465 ± 0.096	0.161 ± 0.055
Nonlinear‐SVR	0.103 ± 0.037	−0.013 ± 0.011	−0.027 ± 0.028	−0.064 ± 0.045	0.102 ± 0.086	−0.014 ± 0.053	0.066 ± 0.043	0.024 ± 0.036
RF	0.371 ± 0.076	0.195 ± 0.112	0.167 ± 0.271	0.152 ± 0.055	0.338 ± 0.089	0.559 ± 0.075	0.279 ± 0.043	0.097 ± 0.060
RMSE (unit: [mmHg])								
MRA	8.94 ± 0.58	10.08 ± 0.66	9.58 ± 0.48	9.72 ± 0.91	14.89 ± 0.60	14.92 ± 1.98	10.28 ± 1.25	8.16 ± 1.09
Linear‐SVR	9.23 ± 1.64	11.03 ± 0.43	9.67 ± 0.71	11.10 ± 0.24	15.16 ± 0.31	15.81 ± 3.19	10.81 ± 0.68	8.61 ± 0.37
Nonlinear‐SVR	13.18 ± 1.34	13.18 ± 0.30	11.57 ± 1.06	13.50 ± 0.81	19.10 ± 0.46	23.53 ± 0.61	14.28 ± 0.58	9.26 ± 0.44
RF	10.92 ± 0.54	11.47 ± 1.10	10.14 ± 1.21	12.06 ± 1.48	16.52 ± 1.49	15.42 ± 0.86	12.56 ± 1.35	8.92 ± 0.47
Relative values								
Coefficient of determination								
MRA	0.205 ± 0.111	0.312 ± 0.021	0.305 ± 0.024	0.420 ± 0.082	0.414 ± 0.151	0.600 ± 0.141	0.445 ± 0.116	0.149 ± 0.070
Linear‐SVR	0.231 ± 0.072	0.366 ± 0.092	0.258 ± 0.072	0.407 ± 0.036	0.379 ± 0.038	0.568 ± 0.062	0.481 ± 0.023	0.127 ± 0.128
Nonlinear‐SVR	−0.047 ± 0.083	0.019 ± 0.076	−0.026 ± 0.071	0.066 ± 0.033	−0.001 ± 0.037	0.045 ± 0.065	0.150 ± 0.043	−0.033 ± 0.054
RF	0.243 ± 0.050	0.217 ± 0.160	0.254 ± 0.082	0.233 ± 0.106	0.508 ± 0.059	0.502 ± 0.101	0.439 ± 0.076	0.077 ± 0.093
RMSE (unit: [mmHg])								
MRA	12.28 ± 0.37	10.90 ± 0.34	9.56 ± 0.84	9.89 ± 1.01	14.78 ± 0.87	14.69 ± 3.19	10.77 ± 0.80	8.50 ± 1.17
Linear‐SVR	12.19 ± 1.66	10.38 ± 0.98	9.82 ± 1.19	10.12 ± 0.28	15.94 ± 0.17	15.26 ± 1.40	10.77 ± 0.43	8.66 ± 1.19
Nonlinear‐SVR	13.63 ± 2.55	12.98 ± 1.02	11.50 ± 1.79	12.65 ± 0.81	20.27 ± 1.08	22.62 ± 2.76	13.71 ± 1.99	9.39 ± 0.89
RF	12.02 ± 2.07	11.52 ± 1.26	9.86 ± 1.21	11.47 ± 0.41	14.19 ± 1.16	16.31 ± 2.91	11.18 ± 0.68	8.98 ± 0.78

First, a comparison of the generalization performance between regression models showed that, for both absolute values and relative values, using MRA as the regression model resulted in a higher average coefficient of determination and a lower RMSE across all subjects. On the other hand, when using nonlinear SVR as the regression model, the average coefficient of determination was lower, and the RMSE tended to be higher.

Next, under the condition where MRA, the highest performing regression model, was used, a comparison of generalization performance between the conditions using absolute values and relative values was made. It was confirmed using absolute values resulted in a higher average coefficient of determination and a lower RMSE across all participants.

Next, we compared the generalization performance between subjects under the condition where the absolute value with the highest generalization performance among the regression models was used, and MRA was employed as the regression model. The results showed that Subjects A and F had high coefficients of determination and low RMSE, whereas Subjects C and H had low coefficients of determination.

Regarding the accuracy of traditional blood pressure monitoring, the measurement error of blood pressure in dialysis support systems has been reported to be within ± 5 mmHg compared with the auscultatory method. The minimum RMSE between the estimated blood pressure and the reference blood pressure shown in Table 4 is approximately 8 mmHg, which is inferior to the measurement accuracy achieved using the dialysis support system.

**Figure 3:**
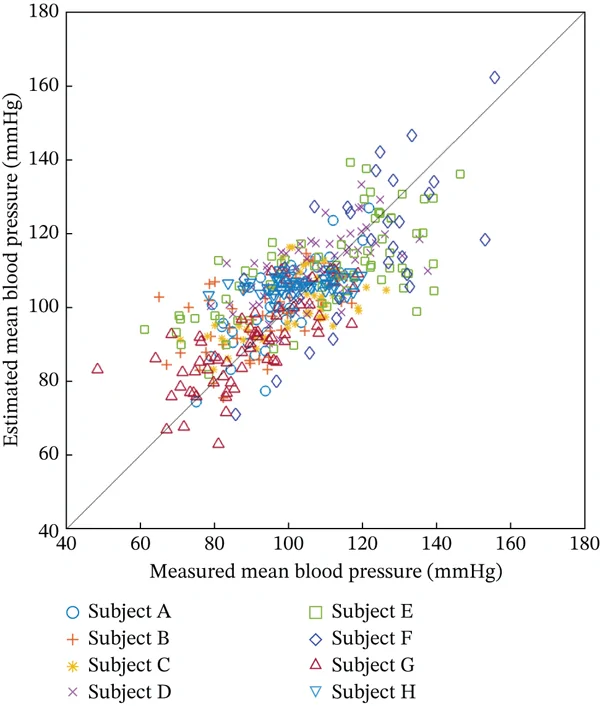
Estimated mean blood pressure plotted against corresponding measured values when applying absolute value to MRA, which demonstrates the highest performance. The results for each participant are plotted using different symbols.

Figure 3 shows a scatter plot of the estimated mean blood pressure plotted against the corresponding measured values when applying absolute value to MRA, which demonstrated the highest performance. This scatter plot represents the results of the cross‐validation with the highest coefficient of determination among the three cross‐validations. The results for each participant are plotted using different symbols.

### 3.2. Evaluation of Explanatory Variables Contributing to Blood Pressure Estimation

Table 5 lists the Top 3 explanatory variables with the highest importance in terms of absolute values and relative values, as determined during cross‐validation with the highest generalization performance using the MRA model. “ROI_hue” shown in the absolute value results represents the absolute value of the hue in the ROI. For example, “Nose_a ^∗^” indicates the absolute value of the a ^∗^ in the nose region. On the other hand, “ROI (A)_ROI(B)_hue” shown in the relative value results represents the difference in hue between ROI (A) and ROI (B). For instance, “Nose_Lip_a ^∗^” indicates the difference between the a ^∗^ value of the nose and that of the lip. If the value is positive, it means that the a ^∗^ value of the nose is larger than that of the forehead. The results indicate that the facial hue components of the a ^∗^ and b ^∗^ values were used as explanatory variables contributing to blood pressure estimation in all subjects. For Subject A, who demonstrated good generalization performance, the absolute value results showed that the hue components of the left infraorbital and nose areas were used as explanatory variables for blood pressure estimation, and even in the relative value condition, the relative hue of the left infraorbital remained important. On the other hand, for Subject D, who also exhibited good generalization performance in blood pressure estimation, the hue components of the nose and forehead were used as explanatory variables, and in the results with relative values, the relative hue component of the nose was used as an explanatory variable. These findings suggest that there are individual differences in the facial regions contributing to blood pressure estimation.

**Table 5:** Top 3 explanatory variables with the highest importance. “ROI_hue” shown in the absolute value results represents the absolute value of the hue in the ROI. “ROI (A)_ROI(B)_hue” shown in the relative value results represents the difference in hue between ROI (A) and ROI (B). *RC* represents the regression coefficient.

Subject	Explanatory variables		
Absolute values			
A	Left infraorbital_a ^∗^ (*R* *C* = −14.26)	Left infraorbital_b ^∗^ (*R* *C* = 5.67)	Nose_b ^∗^ (*R* *C* = −3.52)
B	Nose_b ^∗^ (*R* *C* = 9.44)	Right infraorbital_a ^∗^ (*R* *C* = −7.69)	Forehead_G (*R* *C* = −6.32)
C	Forehead_a ^∗^ (*R* *C* = −5.77)	Nose_b ^∗^ (*R* *C* = −4.29)	Lip_a ^∗^ (*R* *C* = 3.93)
D	Forehead_a ^∗^ (*R* *C* = 20.11)	Nose_b ^∗^ (*R* *C* = 9.13)	Nose_a∗ (*R* *C* = −8.96)
E	Right infraorbital_a ^∗^ (*R* *C* = –67.99)	Right cheek_b∗ (*R* *C* = 59.84)	Right cheek_B (*R* *C* = 40.84)
F	Left infraorbital_a ^∗^ (*R* *C* = –18.43)	Right infraorbital_a ^∗^ (*R* *C* = 8.61)	Lip_a ^∗^ (*R* *C* = 6.44)
G	Right infraorbital_a ^∗^ (*R* *C* = −4.85)	Left infraorbital_b ^∗^ (*R* *C* = −4.60)	Entire face_b ^∗^ (*R* *C* = 3.97)
H	Forehead_a ^∗^ (*R* *C* = −8.12)	Right infraorbital_a ^∗^ (*R* *C* = 3.40)	Right cheek_b ^∗^ (*R* *C* = −1.93)
Relative values			
A	Left infraorbital_Left cheek_a ^∗^ (*R* *C* = −9.61)	Left infraorbital_Entire face_b ^∗^ (*R* *C* = 6.41)	Left cheek_Forehead_R (*R* *C* = −3.22)
B	Nose_Forehead_b ^∗^ (*R* *C* = 7.20)	Left infraorbital_Left cheek_b ^∗^ (*R* *C* = 6.64)	Left infraorbital_Forehead_a ^∗^ (*R* *C* = 5.84)
C	Right cheek_Lip_a ^∗^ (*R* *C* = −6.29)	Left cheek_Forehead_a ^∗^ (*R* *C* = 2.50)	Left infraorbital_Lip_B (*R* *C* = −0.59)
D	Entire face_Forehead_a ^∗^ (*R* *C* = −11.29)	Nose_Forehead_a ^∗^ (*R* *C* = −9.54)	Nose_Forehead_b ^∗^ (*R* *C* = 6.08)
E	Right infraorbital_Nose_b ^∗^ (*R* *C* = −29.63)	Right infraorbital_Nose_B (*R* *C* = −24.64)	Left cheek_Nose_b ^∗^ (*R* *C* = −18.60)
F	Left infraorbital_Right infraorbital_a ^∗^ (*R* *C* = −5.92)	Left cheek_Nose_b ^∗^ (*R* *C* = 5.45)	Left infraorbital_Entire face_b ^∗^ (*R* *C* = −4.15)
G	Right infraorbital_Entire face_b ^∗^ (*R* *C* = −5.61)	Nose_Lip_a ^∗^ (*R* *C* = −3.90)	Right cheek_Entire face_a ^∗^ (*R* *C* = −3.14)
H	Right cheek_Entire face_a ^∗^ (*R* *C* = 14.50)	Right infraorbital_Lip_a ^∗^ (*R* *C* = 7.35)	Right cheek_Lip_R (*R* *C* = −4.74)

## 4. Discussion

This study revealed the following four key findings. These findings suggest that constructing individual models using linear modeling based on the absolute values of facial hues is useful for blood pressure sensing in hemodialysis patients.

### 4.1. Improved Generalization Performance With MRA

In both the absolute and relative value conditions, using MRA as the regression model achieved the highest generalization performance. Although Linear‐SVR and RF also showed relatively high performance, Nonlinear‐SVR exhibited lower performance. One possible reason for this result is the partially linear relationship between facial hue and blood pressure. When such a linear relationship exists, MRA and Linear‐SVR can fit the model more effectively, whereas nonlinear models may become excessively complex, leading to errors. Specifically, Nonlinear‐SVR with an RBF kernel relies heavily on hyperparameter settings, and inappropriate configurations may result in overfitting or underfitting [16]. RF, on the other hand, can partially capture linear signals through decision trees, which allows it to model even somewhat linear relationships between facial hue and blood pressure [17].

### 4.2. Superiority of Absolute Values

Generalization performance was higher when using absolute values compared with relative values. Relative values were used to extract hue components caused by changes in skin blood flow due to blood pressure variations. However, since the reference hue also fluctuated alongside these changes, it became difficult to capture features attributable solely to blood pressure changes. In contrast, using absolute values mitigated the influence of the reference, thereby improving blood pressure estimation accuracy.

Several previous studies have estimated blood pressure using remote photoplethysmography (rPPG), which extracts pulse waveforms from facial videos by tracking subtle color changes over time [18–20]. These methods have achieved notable accuracy (e.g., MAE ≈ 8–11 mmHg) by leveraging temporal information over video sequences of 15–60 s. However, rPPG‐based approaches generally require high‐frame‐rate video acquisition under stable lighting conditions and minimal subject movement, which may be difficult to achieve in clinical environments such as dialysis units.

In contrast, our research group has explored an alternative direction: blood pressure estimation using static facial images. In our previous work [11], spatial features were extracted from visible and infrared facial images and processed using convolutional neural networks. Although the method achieved reasonable RMSE values, it suffered from poor correlation coefficients, especially with visible‐light images, due to the model′s sensitivity to irrelevant spatial features such as blinking or facial muscle activity. The present study further simplifies and strengthens this approach by using hue‐based features extracted from manually defined ROIs, combined with interpretable regression models. Notably, our method requires only a single RGB image per measurement and does not depend on temporal dynamics. This allows for rapid, low‐cost implementation with greater robustness to motion and lighting variability, while maintaining comparable accuracy (RMSE 8–13 mmHg, *r* = 0.2–0.6) in dialysis patients. The ability to track BP trends with such a simple input modality makes our method highly promising for real‐time monitoring in clinical settings.

### 4.3. Individual Differences in Generalization Performance

There were individual differences in generalization performance among participants, attributable to two primary factors: The first factor is gender differences. Males exhibited slightly better generalization performance than females (coefficient of determination: males 0.451, females 0.407; RMSE: males 10.65 mmHg, females 11.11 mmHg). This difference may be due to the difficulty in detecting hue variations in females due to makeup application. The second factor is the magnitude of blood pressure fluctuations during hemodialysis. Participants with large blood pressure fluctuations (Subjects A and F) showed high generalization performance, whereas those with small fluctuations (Subjects C and H) demonstrated lower performance. These findings suggest that the generalization performance of blood pressure estimation depends on the extent of blood pressure fluctuations. The overall RMSE for the proposed method was approximately 8 mmHg, which is slightly higher than the typical error margin (± 5 mmHg) for cuff‐based blood pressure monitors. However, our method provides continuous and noncontact monitoring, which is particularly valuable in clinical settings such as dialysis. This trade‐off between absolute accuracy and continuous, real‐time monitoring capability may justify the use of this technique in scenarios where trend tracking and early detection are prioritized over exact numerical values.

### 4.4. Individual Differences in the Hue Regions Contributing to Explanatory Variables

In the absolute value condition, differences in the contributing hue components were observed between Subjects A and F, who exhibited high estimation performance. For instance, in Subject A, the regression coefficient of the a ^∗^ value for the left infraorbital was negative, whereas in Subject F, the b ^∗^ value had a negative regression coefficient. This indicates that with rising blood pressure, the hue of the left infraorbital changed to yellow‐green for Subject A and to blue‐green for Subject F. It is known that individual differences in facial vascular structure and skin thickness exist [21, 22], and these differences likely contribute to the variation in the hue regions that influence blood pressure estimation. In addition, factors such as skin pigmentation and the depth and density of subcutaneous capillaries may also affect how blood flow variations are reflected in facial hue [23]. These physiological differences can result in subject‐specific patterns of hue change, underscoring the need for personalized modeling in noncontact blood pressure estimation.

### 4.5. Study Limitations

This study has several limitations. First, the study included only eight hemodialysis patients from a single clinical facility. Although multiple dialysis sessions were analyzed for each participant, the small number of participants limits the generalizability of the findings and prevents a sufficiently robust evaluation of the effects of demographic and physiological factors, including age, sex, skin pigmentation, makeup use, vascular structure, and skin thickness. Second, the number of measurement sessions and the range of blood pressure fluctuations differed among participants, which may have affected the individual model performance. Third, facial color features are sensitive to imaging conditions, including ambient illumination, color temperature, automatic exposure, white balance, camera gain, facial orientation, shading, and specular reflection. Because no explicit illumination or color correction was applied, variations in these factors may have influenced the extracted RGB and L ^∗^a ^∗^b ^∗^ features and the resulting model performance. Fourth, the explanatory variables were not standardized or normalized before model construction. Therefore, the magnitudes of the regression coefficients presented in Table 5 may have been affected not only by the physiological relevance of each facial color feature but also by differences in feature scale. Accordingly, comparisons of regression coefficient magnitudes across different color components should be interpreted with caution. Fifth, the machine learning models were evaluated using default hyperparameters without systematic optimization. In particular, the relatively low performance of Nonlinear‐SVR may partly reflect the use of nonoptimized parameter settings. Sixth, reference blood pressure was measured intermittently at approximately 15‐min intervals, and facial hue information was selected from images acquired within 5 min before or after each blood pressure measurement. This temporal mismatch may have introduced measurement uncertainty. Finally, only eight predefined facial regions were analyzed, and potentially informative regions, such as the upper orbital area and inner canthus, were not included.

### 4.6. Future Research Directions

Future studies should validate the proposed approach using a larger and more diverse cohort collected from multiple clinical facilities. Increasing the number of participants and repeated measurement sessions will enable statistical evaluation of the effects of age, sex, skin pigmentation, makeup use, and individual vascular characteristics. Image acquisition protocols should also be standardized, and illumination correction, color calibration, image‐quality assessment using the L ^∗^ component, and robust preprocessing methods should be introduced to reduce the influence of environmental and imaging conditions. Furthermore, systematic hyperparameter optimization and feature standardization should be performed to improve model performance and reproducibility. The use of continuous reference blood pressure monitoring would allow more precise temporal alignment between facial hue changes and hemodynamic fluctuations. Future studies should also evaluate additional facial regions and investigate multimodal approaches combining visible facial hue with near‐infrared or thermal imaging. Ultimately, these developments may contribute to a clinically applicable noncontact system for continuous blood pressure trend monitoring and early detection of intradialytic hypotension.

Recent studies have also demonstrated the clinical potential of near‐infrared spectroscopy (NIRS) for detecting cerebral hypoperfusion through noninvasive optical monitoring of cerebral oxygenation [24]. Such approaches have shown promise for identifying hemodynamic deterioration and related clinical outcomes, including orthostatic cerebral hypoperfusion and fall risk in older adults. Although NIRS and the proposed facial hue analysis evaluate different physiological signals, both approaches share the common goal of noninvasive optical monitoring of circulatory status. Future studies should therefore investigate whether facial hue analysis can complement NIRS‐based monitoring to provide more comprehensive assessment of systemic and cerebral hemodynamic changes.

## 5. Conclusion

This study focused on facial visible images of hemodialysis patients to identify specific regions where hue components fluctuate in response to changes in blood flow. We extracted facial hue features from these regions and evaluated their relationship with blood pressure fluctuations. Individualized models for blood pressure estimation were then constructed using these features. The results demonstrated that applying a linear regression model to the absolute values of facial hue information improved generalization performance across participants. Furthermore, the estimation performance varied between individuals, influenced by factors such as gender and the magnitude of blood pressure fluctuations. The regions of the face contributing most to the estimation also differed by subject, suggesting individual variation in vascular structure and skin characteristics. These findings indicate that personalized models based on facial hue components have potential for noncontact blood pressure monitoring in hemodialysis settings.

Future research should investigate the effects of gender and age, explore hybrid approaches combining facial hue with skin temperature from infrared imaging, and expand the set of facial regions analyzed. Furthermore, it is necessary to evaluate the robustness of the proposed method under varied ambient lighting conditions. Future development should consider integrating compensation mechanisms or multimodal sensing strategies, such as combining visible and infrared imaging, to ensure reliable blood pressure estimation in real‐world environments.

If this study is realized, it would enable the early detection of intradialytic hypotension, which can occur suddenly during hemodialysis, potentially improving the patient′s quality of life (QOL). Furthermore, in our previous research, we developed physiological monitoring technologies based on facial color and skin temperature to estimate health conditions and stress levels [12, 25]. Therefore, the technology developed in this study could be applied to monitoring other health conditions, such as stress monitoring.

Although a commercial RGB camera was used in this study, the proposed approach demonstrates a novel sensing principle by extracting physiologically relevant features—facial hue components—from optical images. This technique, by serving as a software‐based sensing layer, enables the transformation of commonly available imaging devices into functional noncontact blood pressure sensors. Therefore, our work can be seen as a foundation for developing low‐cost, scalable, and continuous blood pressure monitoring systems, which aligns with the broader vision of remote and wearable healthcare sensing technologies.

## Author Contributions

Kosuke Oiwa conducted the data analysis and interpretation, and was responsible for drafting the manuscript. Takehiro Okama contributed to data acquisition and analysis, and reviewed the manuscript critically. Satoshi Suzuki supervised the overall study and contributed to the critical revision of the manuscript. Yoshitaka Maeda provided the experimental equipment, contributed to the data analysis, and participated in manuscript revision. Akio Nozawa proposed the algorithm used in this study and assisted in the critical review of the manuscript.

## Funding

This study was supported by the Japan Society for the Promotion of Science (10.13039/501100001691) (23K11954).

## Disclosure

The funder had no role in the study design, data collection and analysis, preparation of the manuscript, interpretation, writing of the manuscript, or the decision to submit the article for publication. The authors take full responsibility for the content of this manuscript.

## Conflicts of Interest

The authors declare no conflicts of interest.

## Data Availability

The data that support the findings of this study are available on request from the corresponding author. The data are not publicly available due to privacy or ethical restrictions.
